# Intramuscular [^18^F]F-FDG Administration for Successful PET Imaging of Golden Hamsters in a Maximum Containment Laboratory Setting

**DOI:** 10.3390/v14112492

**Published:** 2022-11-11

**Authors:** Hui Wang, Jurgen Seidel, Christopher Bartos, Russell Byrum, Philip J. Sayre, Kurt Cooper, Yu Cong, Dong-Yun Kim, Claudia Calcagno, Jens H. Kuhn, Anya Crane, Jiro Wada, Reed F. Johnson, Dima A. Hammoud, Ji Hyun Lee

**Affiliations:** 1 Integrated Research Facility at Fort Detrick, Division of Clinical Research, National Institute of Allergy and Infectious Diseases, National Institutes of Health, Fort Detrick, Frederick, MD 21702, USA; 2 Office of Biostatistics Research, National Heart, Lung and Blood Institute, National Institutes of Health, Bethesda, MD 20892, USA; 3 Center for Infectious Disease Imaging, Radiology and Imaging Sciences, Clinical Center, National Institutes of Health, Bethesda, MD 20892, USA; 4 Radiology and Imaging Sciences, Clinical Center, National Institute of Health, Bethesda, MD 20892, USA

**Keywords:** infectious diseases, imaging, [^18^F]F-FDG PET, administration route, biosafety level 4, intramuscular, intravenous, maximum containment, small-animal PET, hamster

## Abstract

Positron emission tomography (PET) is becoming an important tool for the investigation of emerging infectious diseases in animal models. Usually, PET imaging is performed after intravenous (IV) radiotracer administration. However, IV injections are difficult to perform in some small animals, such as golden hamsters. This challenge is particularly evident in longitudinal imaging studies, and even more so in maximum containment settings used to study high-consequence pathogens. We propose the use of intramuscular (IM) administration of 2-deoxy-2[^18^F]fluoro-D-glucose ([^18^F]F-FDG) for PET imaging of hamsters in a biosafety level 4 (BSL-4) laboratory setting. After [^18^F]F-FDG administration via IM or IV (through surgically implanted vascular access ports), eight hamsters underwent static or dynamic PET scans. Time–activity curves (TACs) and standardized uptake values (SUVs) in major regions of interest (ROIs) were used to compare the two injection routes. Immediately after injection, TACs differed between the two routes. At 60 min post-injection, [^18^F]F-FDG activity for both routes reached a plateau in most ROIs except the brain, with higher accumulation in the liver, lungs, brain, and nasal cavities observed in the IM group. IM delivery of [^18^F]F-FDG is an easy, safe, and reliable alternative for longitudinal PET imaging of hamsters in a BSL-4 laboratory setting.

## 1. Introduction

Small-animal 2-deoxy-2[^18^F]fluoro-D-glucose ([^18^F]F-FDG) positron emission tomography (PET) is becoming an important tool in the characterization of emerging infectious diseases [1,2,3]. However, PET imaging of small-animal models commonly used in infectious disease research, such as domesticated guinea pigs (*Cavia porcellus* (Linnaeus, 1758)) and golden hamsters (*Mesocricetus auratus* (Waterhouse, 1839)), presents considerable challenges.

Precise and consistent administration of [^18^F]F-FDG is necessary for accurate quantitative PET analysis. However, intravenous (IV) injections are difficult to perform in small rodents with absent or inaccessible tail veins. In fact, the rate of extravasation after IV radiotracer injection in these animals is higher than the 15% found in clinical settings [4]. Cannulation of cephalic and penile veins or intracardial injections have been described as alternatives to tail vein injections [5,6,7], but these routes are also technically challenging. Surgically implanted vascular access ports (VAPs) have shown some success [2]; however, VAP port displacement because of animal growth often precludes their use in longer longitudinal studies. These technical challenges are further exacerbated in maximum containment (U.S.: biosafety level 4 [BSL-4]) laboratories used to study high-consequence pathogens because of multiple layers of personal protective equipment (PPE) that have to be worn by the experimenters and the specific logistics and safety requirements needed to run experiments in this setting [8].

To increase the feasibility of PET imaging of small-animal models of infectious diseases, there is a need for alternatives to IV radiotracer administration. In this study, we analyzed major organs (brain, lungs, liver, kidneys, myocardium, and spleen) as well as nasal cavities, muscle, and bone marrow for [^18^F]F-FDG post-injection kinetics and uptake after IV (through VAPs) or intramuscular (IM) administration in two groups of golden hamsters by performing serial PET scans in a BSL-4 laboratory. Our results indicate that IM [^18^F]F-FDG administration is an easy, efficient, and safe alternative to IV for successful PET imaging in this animal model.

## 2. Materials and Methods

### 2.1. Study Design

All experiments were conducted in a BSL-4 Laboratory at the National Institutes of Health (NIH) National Institute of Allergy and Infectious Diseases (NIAID) Division of Clinical Research (DCR) Integrated Research Facility at Fort Detrick (IRF-Frederick) in Frederick, MD, USA. All animal experiments were performed in accordance with animal study protocols approved by the NIAID DCR Animal Care and Use Committee.

Eight hamsters (five males, three females; median age 35.5 (interquartile range 7.6–36.0) weeks, weight 132.5 [119–167.5] g) were purchased from Charles River Laboratories (Wilmington, MA, USA). The hamsters were individually housed with ad libitum access to food and water in an environment with temperatures of 21–23 °C, relative humidity 40–60%, and a 12 h light/12 h dark cycle.

**Figure 1:**
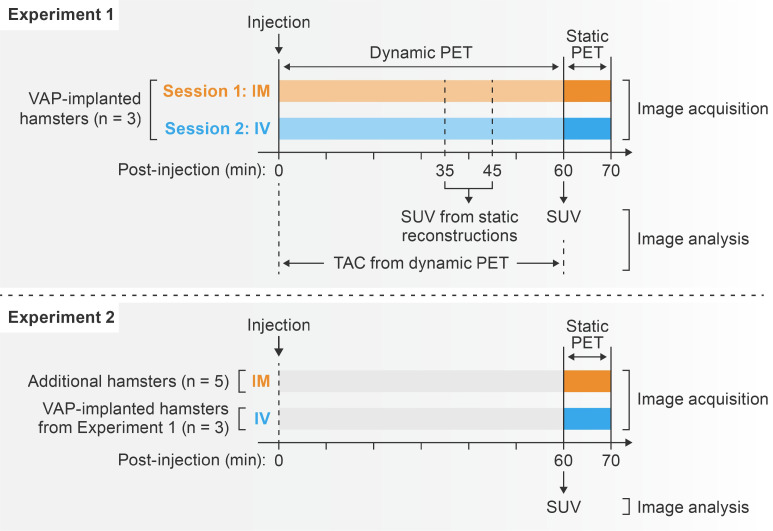
[^18^F]F-FDG PET schedule. [^18^F]F-FDG, 2-deoxy-2-[^18^F]fluoro-D-glucose; PET, positron emission tomography; VAP, vascular access port; IM, intramuscular; IV, intravenous.

The study was divided into two sets of experiments (Figure 1). In Experiment 1, three VAP-implanted hamsters underwent a 60-min dynamic PET imaging session right after either IV or IM [^18^F]F-FDG administration on two separate days. Dynamic scans were followed by 10-min static PET imaging at 60 min post-injection. Time–activity curves (TACs) were used to investigate differences in the temporal distribution of [^18^F]F-FDG between the IV and IM routes. Static reconstructions at 30–40 min and 40–50 min post-injection were created from the same set of dynamic data and, together with the static scans acquired at 50–60 min post-injection, were used to examine [^18^F]F-FDG distribution in major areas at three representative time points (35, 45, and 55 min post-injection). Since TACs indicated that radiotracer uptake plateaued in most organs except the brain at around 60 min post-injection for both administration routes, we performed a second experiment to investigate [^18^F]F-FDG organ distribution in a larger set of hamsters at this time point. For this experiment, Experiment 2, we included static scans acquired at 50–60 min after IV injection from the same VAP-implanted hamsters (all 7.6 weeks old at the time of imaging) from Experiment 1 and static scans acquired at 50–60 min after IM injection from five additional hamsters (median age 36.0 weeks, interquartile range 35.6–38.2 weeks old). Standardized uptake values (SUVs) of major areas were compared between the IM and IV groups.

### 2.2. Image Acquisition

[^18^F]F-FDG was purchased from Cardinal Health (Beltsville, MD, USA) and diluted with sodium chloride (USP 0.9%, Fresenius Kabi USA LLC, Lake Zurich, IL, USA) to a concentration of 110–185 MBq/mL. Hamsters were anesthetized with 2–5% isoflurane in oxygen throughout the imaging session, with vital signs continuously monitored using a small-animal monitoring and gating system (model 1030, SA Instruments, Inc., Stony Brook, NY, USA). All hamsters were imaged on an MRS*PET/CT 120 scanner (MR Solutions, Guildford, UK), located inside a BSL-4 laboratory.

After [^18^F]F-FDG injection, either dynamic or static PET imaging was performed as described in the study design (Figure 1), followed by whole-body computed tomography (CT) scans for attenuation correction. For IM administration, the volume of [^18^F]F-FDG was limited to 0.1 mL per injection site. PET data were acquired in list mode and reconstructed using a three-dimensional ordered subsets expectation-maximization algorithm (4 iterations and 32 subsets). Corrections for photon attenuation, Compton scattering, random coincidence, and dead-time losses were applied.

### 2.3. Correction for [^18^F]F-FDG Dose Retention

All PET scans acquired after IM administration showed visible radiotracer retention at the injection site. To account for this, the [^18^F]F-FDG effective dose delivered to hamsters was corrected using an adapted method [9]. First, two regions of interest (ROIs), encompassing the whole body and the residual radioactivity at the retention site were drawn. The total lesion glycolysis (TLG) of the ROIs, the product of mean SUV (SUV_mean_), and volume of the ROI were recorded. Then, the corrected dose was calculated using Equation (1), in which TLG_WB = TLG of whole-body ROI; TLG_R = TLG of residual ROI.

(1) $$Corrected\;dose\left(mCi\right)=Initial\;FDG\;dose\;\left(mCi\right)*\frac{TLG\_WB-TLG\_R}{TLG\_WB}$$

### 2.4. Image Analyses

Image analyses were performed using MIM software version 7.1.2 (Cleveland, OH, USA). For static scans, spherical ROIs, 2–5 mm in diameter, were manually placed in major organs (brain, lungs, liver, kidneys, myocardium, and spleen) as well as nasal cavities, muscle, and bone marrow using CT images as anatomical references. TACs were generated for dynamic scans, and SUV_mean_ of ROIs was recorded for static acquisitions/reconstructions.

### 2.5. Statistical Analyses

SUVs are shown as mean ± standard deviation (SD). Statistical analyses were performed using Prism v.9 (GraphPad, San Diego, CA, USA). Two-way analysis of variance (ANOVA) with Tukey’s test for multiple comparison was used to determine the effect of injection routes on the static SUV values. Injection route, uptake time, interaction between route and uptake time, and individual hamster were considered as sources of variation. F ratio was computed to determine whether the between-route variances are significantly greater than would be expected if there were no route effect. For dynamic imaging, time to peak (TTP) values were calculated using an area-under-the-curve (AUC) analysis of all TACs. Differences in SUV_mean_ across areas analyzed at 60 min post-injection between the IM and IV groups were compared using independent t-tests. An adjusted *p*-value of less than 0.05 was considered statistically significant.

## 3. Results

Information on experimental groups and experiment schedule is provided in Figure 1.

### 3.1. [^18^F]F-FDG Dose Retention in IM Group

The average [^18^F]F-FDG injected dose was 32.3 ± 2.7 MBq per hamster. In the IM group, the [^18^F]F-FDG radioactivity retention was clearly visible at the injection site in all PET images and accounted for 8.73 ± 3.38% of whole-body radioactivity (Video S1).

### 3.2. [^18^F]F-FDG Kinetics

**Figure 2:**
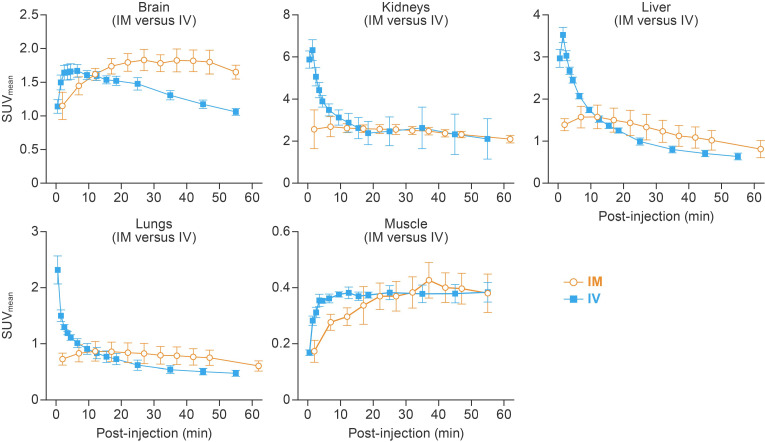
Temporal and spatial distribution of [^18^F]F-FDG after IM and IV injections. Dynamic PET scans were performed 0–55 min post-injection for IM and 2–62 min for the IV route. Mean time–activity curve of the brain, lungs, liver, kidneys, and muscle are presented. Error bars represent one SD. [^18^F]F-FDG, 2-deoxy-2-[^18^F]fluoro-D-glucose; IV, intravenous; IM, intramuscular; SUV_mean_, mean standardized uptake value; PET, positron emission tomography; SD, standard deviation.

TACs showed distinct [^18^F]F-FDG kinetics for IM and IV routes (Figure 2). Following IV administration, [^18^F]F-FDG radioactivity peaked at 5.2 ± 1.2 min post-injection in the brain and then decreased gradually afterwards. After IM administration, the time to peak [^18^F]F-FDG radioactivity in the brain was significantly delayed to 36.3 ± 19.0 min post-injection (*p* = 0.047) and remained at a plateau until the end of the scan. The IM group also had a delayed [^18^F]F-FDG peak in the lungs (10.0 ± 7.6 min versus 0.5 ± 0.0 min, *p* = 0.090), liver (7.0 ± 5.0 min versus 1.5 ± 0.0 min, *p* = 0.130), and kidneys (20.3 ± 16.1 min versus 1.2 ± 0.6 min, *p* = 0.108) compared to the IV group, although these differences did not reach statistical significance. TTPs in the muscle were not different between injection methods (42.0 ± 5.0 min for IM versus 40.8 ± 24.5 min for IV). By 60 min post-injection, [^18^F]F-FDG radioactivity in all tested organs and areas reached a plateau for both administration methods.

### 3.3. IM Injection Results in Higher [^18^F]F-FDG Accumulation in Brain and Liver

**Figure 3:**
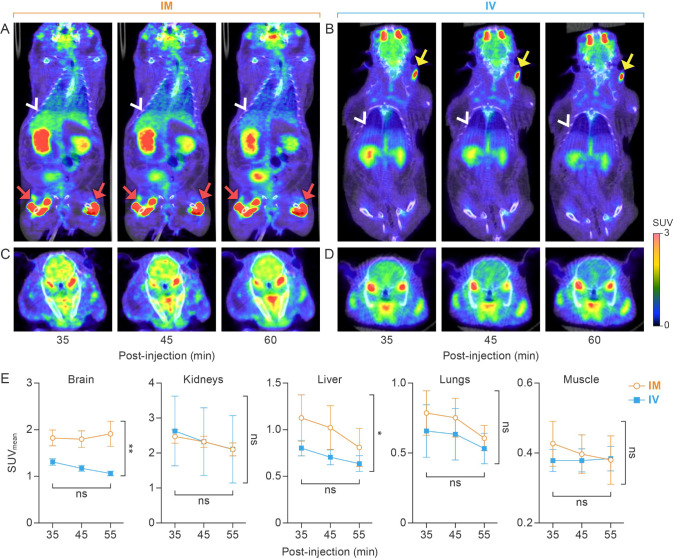
Comparison of [^18^F]F-FDG distribution at different time points after IM or IV administration. (**A**) Coronal section of whole-body PET at 35, 45, and 60 min post-injection via the IM route. White arrowheads point to the liver. Red arrows show [^18^F]F-FDG residual/extravasation representative of that found in the IM group, respectively. (**B**) Coronal section of whole-body PET at 35, 45, and 60 min post-injection via the IV route. White arrowheads point to the liver. Yellow arrows show [^18^F]F-FDG residual/extravasation representative of that found in the IV group. (**C**) Trans-axial section images of the brain at 35, 45, and 60 min post-injection via the IM route. (**D**) Trans-axial section images of the brain at 35, 45, and 60 min post-injection via the IV route. All images are from a single golden hamster that received IM and IV administration on different days. (**E**) Two-way ANOVA indicates that the IM route results in increased [^18^F]F-FDG accumulation in the liver (*p* = 0.03) and brain (*p* = 0.005) as compared to the IV group. [^18^F]F-FDG accumulation in lungs, kidney, and muscle tissue was not statistically different between IM and IV routes. [^18^F]F-FDG, 2-deoxy-2-[^18^F]fluoro-D-glucose; IM, intramuscular; IV, intravenous; PET, positron emission tomography; ANOVA, analysis of variance. *, *p* < 0.05; **, *p* < 0.01.

Two-way ANOVA of static PET reconstructions and scans at 35, 45, and 55 min post-injection demonstrated that IM administration results in higher [^18^F]F-FDG accumulation in the liver (F = 7.5, *p* = 0.03; Figure 3) and brain (F = 18.36, *p* = 0.005; Figure 3), whereas the radiotracer uptake time and individual hamster had no significant effect. The slightly higher [^18^F]F-FDG accumulation in the lungs, observed in the IM group, was attributed to the variability of tracer uptake among hamsters (*p* = 0.008), not due to the injection route or [^18^F]F-FDG uptake time (F = 3.9, *p* = 0.10). SUV_mean_ in the kidneys and muscle were not different between IM and IV groups (Figure 3).

### 3.4. SUV_mean_ at 60 min Post-Injection

**Figure 4:**
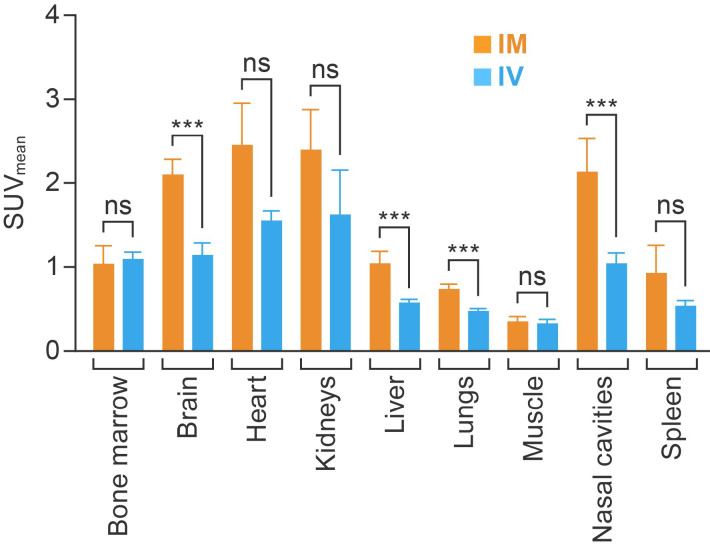
Comparing the average SUV_mean_ for major organs and areas at 60 min after IM (n = 5) and IV (n = 3) injection. Each column represents the group average per administration route. The value in each cell represents the average SUV_mean_. SUV_mean_, mean standardized uptake value; IM, intramuscular; IV, intravenous; ns, not significant; ***, *p* < 0.01.

Further analyses of PET data acquired at 60 min post-injection indicated that [^18^F]F-FDG uptake in bone marrow, myocardium, kidneys, spleen, and muscle were comparable in both the IM and IV groups. However, the IM route resulted in higher [^18^F]F-FDG accumulation compared with the IV route in the brain (2.10 ± 0.18 versus 1.14 ± 0.15, *p* < 0.01), lungs (0.74 ± 0.06 versus 0.58 ± 0.04, *p* < 0.01), liver (1.04 ± 0.15 versus 0.58 ± 0.04, *p* < 0.01), and nasal cavities (2.14 ± 0.39 versus 1.05 ± 0.12, *p* < 0.01) (Figure 4).

## 4. Discussion

Accurate and reliable IV radiotracer administration can be particularly challenging for [^18^F]F-FDG PET imaging of golden hamsters, especially in a BSL-4 laboratory setting. Intrinsic difficulties in accessing veins in this animal model, the need for frequent longitudinal scans to assess the course of infection, and the multiple layers of PPE required in BSL-4 settings negatively impact the success rate of IV administration of [^18^F]F-FDG.

Since hamsters do not have tail veins, surgically implanted VAPs can be used as a venous access for [^18^F]F-FDG delivery. However, VAP displacement during the course of longitudinal experiments can be a relatively common cause of misinjection. Our laboratory has observed a rate of [^18^F]F-FDG extravasation as high as 54.55% (12 extravasations out of 22 IV injections) when using VAPs in hamsters, with some extravasations close to major organs and areas that interfere with SUV measurement (Video S2). In the case of our study, VAP displacement was likely due to the fast growth of young hamsters during the course of the study; this issue may be prevented by using only mature animals. However, some studies may require the use of both young and aged hamsters, depending on susceptibility to pathogens being studied. VAP displacement may also be caused by animals’ daily activities. As a result, it is challenging to ensure successful IV radiotracer administration throughout a longitudinal imaging study that uses this common small-animal model, and these issues likely apply to other small animals with difficult intravenous access.

Intraperitoneal (IP) radiotracer injection is a common alternative route to IV delivery [10], which also presents some significant challenges. First, IP administration is associated with a relatively high error rate (10–25%) due to erroneous placement of the inoculum into a site other than the peritoneal cavity [11,12]. Second, after IP administration, the radiotracer needs to diffuse across the peritoneal membrane and be absorbed through the portal system [13]; a fraction of the radiotracer may pass across the diaphragm through small lacunae into the thoracic lymph [14]. We have also observed higher [^18^F]F-FDG accumulation in the abdomen with IP injection (Figure S1), which could either be caused by erroneous injection or IP diffusion. For these reasons, IP administration of [^18^F]F-FDG may not be suitable for the quantitative assessment of metabolic activity in liver and lymphoid organs (e.g., spleen and lymph nodes), which are of great interest in infectious disease research.

Research on IM administration as an alternative radiotracer delivery route is scarce. One issue with IM radiotracer administration is that the residual [^18^F]F-FDG remaining at the injection site is slowly and continuously absorbed into circulation and therefore makes quantitative PET analyses more complex. The absorption and bioavailability of drugs after IM injection is influenced by the injection site, diluent, solubility of drug, concentration of drug, total surface area of diffusion, and blood flow to injected muscle [15,16]. In our study, we controlled these factors by following a standardized study protocol, which (1) specified the [^18^F]F-FDG concentration, (2) limited the volume of the [^18^F]F-FDG inoculum to 0.1 mL per injection site, (3) directed [^18^F]F-FDG injection to the same location throughout the study, and (4) monitored and maintained vital signs within a stable range. Our rigorous protocol minimized the influence of these factors on [^18^F]F-FDG absorption and bioavailability, as evidenced by a relatively consistent residual activity at the injection sites (8.73 ± 3.38%). In addition, we applied a manual ROI-based [^18^F]F-FDG dose-correction method to all IM group data. However, we observed a higher variability in the IM group, as shown in Figure 2 and Figure 3, which may be due to the continuous absorption of residual [^18^F]F-FDG from the injection site into circulation. Additionally, static PET images were acquired 60 min after IM administration, when [^18^F]F-FDG radioactivity reached a steady state in the major organs and areas. Even though IM administration leads to higher [^18^F]F-FDG accumulation in some organs and areas, the steady-state SUV values allow for comparison of metabolic activities among different groups of hamsters.

Our study has several limitations: First, a minimum of 6 h fasting is generally recommended for [^18^F]F-FDG PET scans. However, hamsters often store food in their cheek pouches until they are ready to consume it, and therefore removing the food tray does not guarantee fasting. Thus, no fasting was performed in this study. Second, it is known that high blood glucose levels affect [^18^F]F-FDG uptake in certain organs/areas. In our study, blood glucose levels were not monitored given the young age and the consistent housing/diet conditions of the hamsters. Third, since the [^18^F]F-FDG radioactivity in the brain does not reach a plateau at 60 min post-injection, caution should be taken when using IM administration route for brain [^18^F]F-FDG PET analysis. Last, the median age for the IV and IM group was 7.6 weeks and 36.0 weeks, respectively. Due to the limited [^18^F]F-FDG PET studies in hamsters, it is not clear if and how the age will affect the [^18^F]F-FDG uptake in the major organs. Age matching should be considered for further investigations.

## 5. Conclusions

Our study demonstrates that the IM route is a safe and efficient alternative to IV [^18^F]F-FDG administration in golden hamsters when a rigorous study protocol, including fixed [^18^F]F-FDG concentration, injection volume, injection site, image acquisition time, scan duration, dose correction, data inclusion/exclusion criteria, and animal-handling procedures, is developed and followed strictly throughout a longitudinal PET imaging study.

## Data Availability

Not applicable.

## Supplementary Materials

The following supporting information can be downloaded at: https://www.mdpi.com/article/10.3390/v14112492/s1, Figure S1: [^18^F]F-FDG uptake in the abdomen; Video S1: [^18^F]F-FDG extravasation in the IV group; Video S2: Residual [^18^F]F-FDG activity at the injection site in the IM group.
