# Supplementary material for: Intramuscular [18F]F-FDG Administration for Successful PET Imaging of Golden Hamsters in a Maximum Containment Laboratory Setting
Source: Viruses. 2022 Nov 11;14(11):2492. doi: 10.3390/v14112492 (PMC9695137; doi:10.3390/v14112492)
Supplement: Supplementary file 1 [file viruses-14-02492-s001.zip › viruses-1974183-supplementary-Figure and video.pdf]

viruses

MDPI

Supplementary Materials

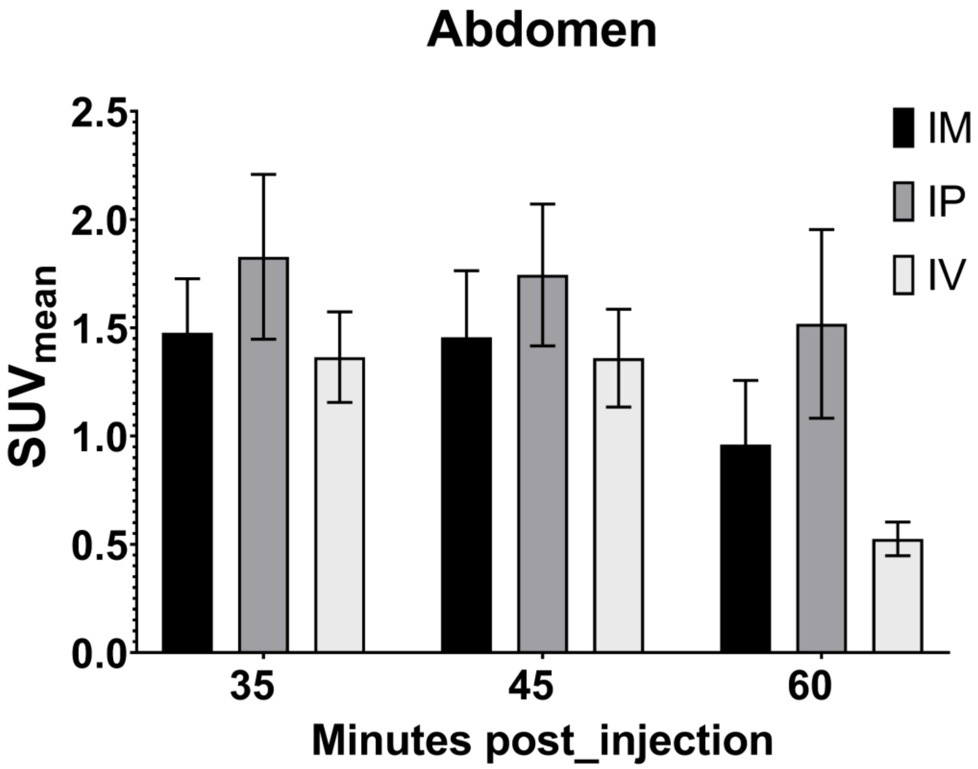

Figure S1. [18F]F-FDG uptake in the abdomen. IP administration is associated with higher [18F]F-FDG uptake in abdomen at 35, 45, and 60 min post-injection, as compared to IV and IM administration routes. IP administration was performed in 6 hamsters (3 males and 3 females; median age 35.9 weeks, interquartile range 35.3-37.6 weeks old). [18F]F-FDG, 2-deoxy-2-[18F]fluoro-D-glucose; SUV_{mean}, mean standardized uptake value at indicated time points post injection; IM, intramuscular; IP, intraperitoneal; IV, intravenous.

Video S1. [18F]F-FDG extravasation in the IV group. The blue contour encircles the whole body and was used to calculate TLG_WB. The yellow contour encircles the extravasated [18F]F-FDG activity. [18F]F-FDG, 2-deoxy-2-[18F]fluoro-D-glucose; TLG, total lesion glycolysis; WB, whole body.

Video S2. Residual [18F]F-FDG activity at the injection site in the IM group. The blue contour encircles the whole body and was used to calculate TLG_WB. The yellow contour encircles the residual [18F]F-FDG activity and was used to calculate TLG_R. [18F]F-FDG, 2-deoxy-2-[18F]fluoro-D-glucose; TLG, total lesion glycolysis; WB, whole body; R, residual.
